# Causal effects of fatty acids on atopic dermatitis: A Mendelian randomization study

**DOI:** 10.3389/fnut.2023.1083455

**Published:** 2023-02-23

**Authors:** Jia-Ying Lin, Liang-Juan Ma, Jin-Ping Yuan, Pei Yu, Bing-Xue Bai

**Affiliations:** Department of Dermatology, The Second Affiliated Hospital of Harbin Medical University, Harbin, Heilongjiang, China

**Keywords:** fatty acids, atopic dermatitis, omega-3 fatty acids, *FADS* gene, Mendelian randomization study

## Abstract

**Background:**

Some evidence suggests abnormalities in fatty acids in patients with atopic dermatitis (AD), and benefits of supplementation with these fatty acids have been reported. However, there is still substantial controversy on the correlation between fatty acids and AD. Therefore, the aim of this study was to determine whether fatty acid levels are causally related to AD using a Mendelian randomization approach.

**Methods:**

We evaluated the data about the fatty acids levels and AD with various methods from Genome-Wide Association Study (GWAS). GWAS results were available both from European ancestry. Mendelian randomization methods were used to analysis the casual inference of fatty acids on AD. MR Egger and MR-PRESSO were used to determine pleiotropy and heterogeneity. Further analysis was conducted using instruments associated with the *FADS* genes to address mechanisms involved. We also used Multivariate MR (MVMR) to show the independent casual inference of omega-3 (n-3) fatty acids on AD.

**Results:**

Mendelian randomization (MR) analysis suggests that n-3 fatty acid levels are associated with a lower risk of AD (n-3 OR_IVW_: 0.92, 95% confidence interval [CI]: 0.87–0.98; *p* = 0.01). Moreover, docosahexaenoic acids (DHA) levels, which is a kind of long-chain, highly unsaturated omega-3 (n-3) fatty acid, and its higher level was associated with a lower risk of AD (DHA ORIVW: 0.91, 95% CI: 0.84–0.98; *p* = 0.02). We ran multivariable MR analysis while controlling for variables within the other types of fatty acids. The effect estimates agreed with the preliminary MR analysis indicating the effect of n-3 fatty acids levels on AD was robust. MR-egger suggest no significant pleiotropy and heterogeneity on genetic instrumental variants. Outliers-corrected MR analyses after controlling horizontal pleiotropy were still robust. The single-SNP analyses revealed that n-3 fatty acids are likely linked to a decreased risk of AD through *FADS* cluster, highlighting the significance of the *FADS* gene in the fatty acids synthesis pathway in the development of AD.

**Conclusion:**

Our studies suggest that n-3 fatty acids may reduce the risk of AD. Risk prediction tools based on n-3 fatty acid levels may be valuable methods for improving AD screening and primary prevention. To reduce the risk of AD, individuals could enhance n-3 fatty acids intake through supplement or diet.

## 1. Introduction

Atopic dermatitis (AD) is a chronic inflammatory skin disease, mainly presented serve itching and recurring eczematous lesions (1). The prevalence of AD is up to 10–20% among adults and children in developed countries (2). It is the most prevalent non-fatal skin disease, imposing a significant psychosocial expense on individuals and their families, and raising the risk of allergic diseases and other immune-mediated inflammations as well as mental health issues. The pathophysiology of AD is complex, involving a strong genetic susceptibility, epithelial barrier dysfunction, immune dysregulation, and abnormalities in the skin microbiome (3). Barrier dysfunction in AD is associated with lipid abnormalities in the stratum corneum, which may be caused by the disturbance between degradation proteases and lipid synthesis enzymes (4). Recent study showed the lack of sebum may develop AD *via* IL-33 signal pathway initiating skin inflammation and mentioned the role of Microbial dysbiosis in AD (5).

Fatty acids are a series of carboxylic acids with an aliphatic chain, saturated or unsaturated, including saturated fatty acids (SFAs), monounsaturated fatty acids (MUFAs) as well as polyunsaturated fatty acids (PUFAs). In either form, fatty acids are an essential energy source of the human diet and an important structural component of cells (6). It has been proved that dietary intake of fatty acids have a relationship with the development of inflammatory and immune system (7).

Essential fatty acids (EFAs) are a type of PUFAs that must be obtained through diet because it cannot be synthesized by human bodies (8). Dermatitis (scaling and dry skin) and increased TEWL are two typical clinical signs of EFAs deficiency (9, 10). EFAs may have beneficial effects in health and in the control of chronic diseases (11). The two types of EFAs include an omega-6 (n-6) fatty acid (linoleic acid) and an omega-3 (n-3) fatty acid (alpha-linolenic acid). EFAs deficiency in the skin is one suspected factor involved in AD (12). An altered stratum corneum lipid composition signifies the importance of ceramides and free fatty acids for the impaired skin barrier function in AD (13). Although studies showing altered PUFA levels in the development of AD, it cannot be ruled out that diets or other social factors result in the reverse causation.

Nutrient supplementation, including fatty acids, can prevent the development of AD or reduce the severity of AD in newborns to children younger than 3 years suggesting the role of fatty acids in AD (14). However, there is no evidence on whether PUFAs have causal effects on AD.

Nowadays many advanced methods to identify causal inference are given rise to use because randomized Controlled Trails (RCTs) are easily influenced by human factors and social environment, which can help to guide whether RCTs are theoretically required. Mendelian randomization (MR) can avoid reverse causation and the interference of weaken confounding factors to address causal interference regarding how exposures affect the different outcomes by using genetic instrumental variants (15). Three principal assumptions should be met. The first hypothesis is that genetic instrumental variants are closely related to exposure factors. The second hypothesis is that genetic instrumental variants should strictly not be related to any confounding factors. The third hypothesis is that genetic instrumental variants affect the outcome only through exposure factors and not through other pathways. The present study applied a two-sample MR approach to figure out whether fatty acids levels are related to AD and which PUFAs are most important.

## 2. Materials and methods

### 2.1. Identifying genetic instruments for the fatty acids

Instruments on circulating levels of n-3 fatty acids, n-6 fatty acids as well as other fatty acids are acquired from a genome-wide association studies (GWASs) (*N* = 114,999) including 6 types of fatty acids in UK Biobank (16, 17).

**Figure 1:**
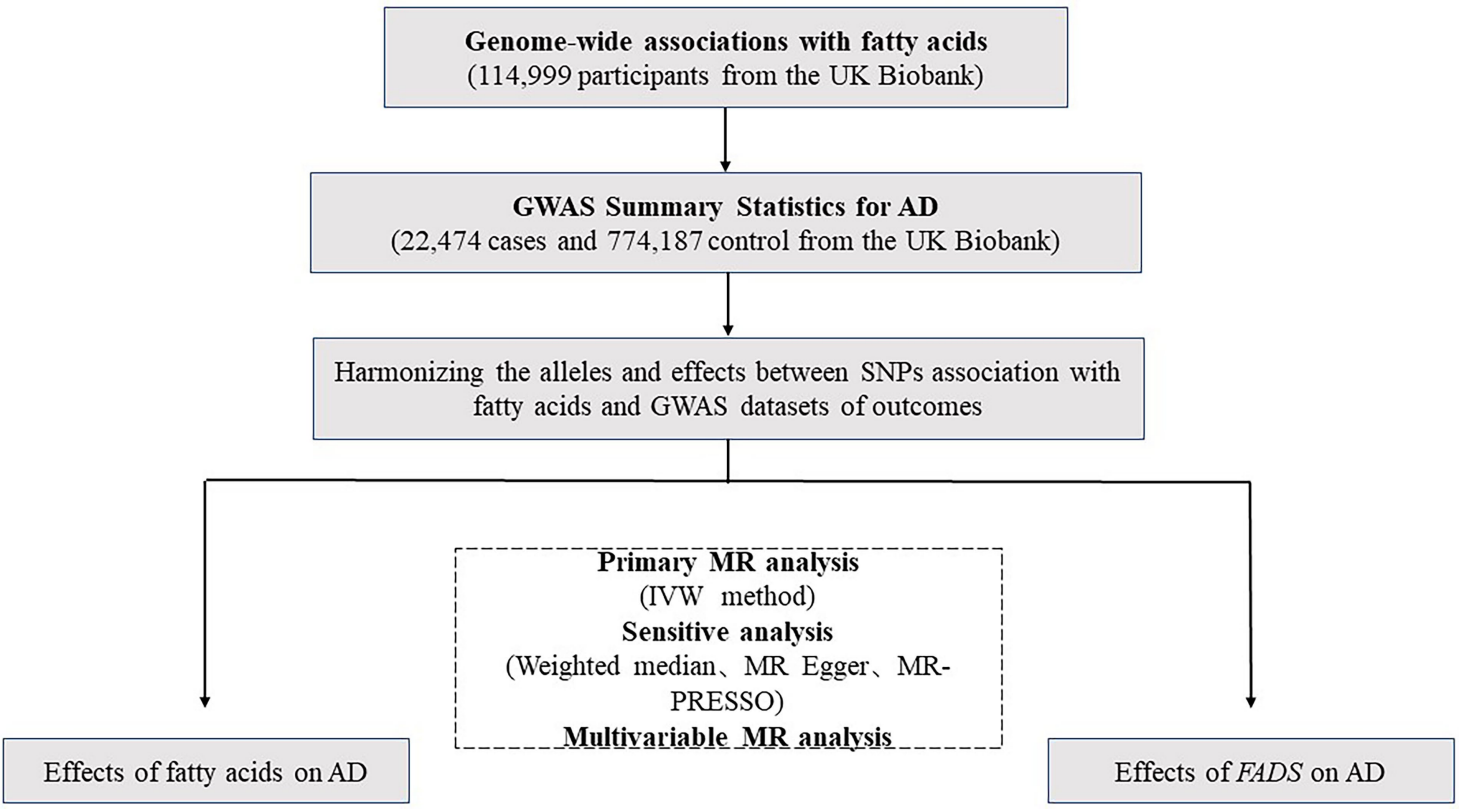
A flowchart of the study.

We primarily selected Single nucleotide polymorphisms (SNPs) with significant evidence of association (*p* ≤ 5e-8), and then we excluded the SNPs whose minor allele frequency ≤ 0.01. TwoSampleMR R package was used to remove instrumental variants with linkage disequilibrium (LD) (R2 > 0.001) (18). Finally, we extracted 48 independent SNPs for n-3 fatty acids, 51 independent SNPs for n-6 fatty acids, 53 independent SNPs for PUFAs, 47 independent SNPs for SFAs and 56 independent SNPs for MUFAs (Figure 1), which were then used to as instruments of fatty acids levels, respectively (see below).

### 2.2. Genome-Wide Association Study of atopic dermatitis

The SNP information that are consistent with each fatty acids instrument SNP was extracted from the AD GWAS (N = 796,661, N_cases_ = 22,474) in the FinnGen study, the Estonian Biobank, and the UK Biobank reported in 2021 (19).

### 2.3. Mendelian randomization analysis

#### 2.3.1. Univariable Mendelian randomization analyses

After harmonizing SNP-fatty acids with SNP-AD data, univariable MR analyses were conducted applying the Two sample MR R package. The inverse variance weighted (IVW) method (15) was mainly applied to evaluate casual effects of fatty acids on AD. Besides, other MR methods were used, including the weighted median (20), weighted mode (21), and MR Egger (22), each of which makes different assumptions about instrument validity.

Therefore, the MR-egger (23) was used to examine pleiotropy, and then we conducted outliers-corrected MR analyses and removed weak or pleiotropic instruments detected by MR-PRESSO (23) to control heterogeneity in these MR estimates.

#### 2.3.2. Multivariable Mendelian randomization analyses

Since most of the fatty acids instruments that are also related with others’ sets, the independent casual effect of n-3 fatty acids was estimated using Multivariable MR (MVMR) (24).

### 2.4. Assessing instrument strength and effect heterogeneity

The variance (R^2^) for genetic instrumental variants was calculated by the following equation:


$$R^{2}=2\times MAF\times \left(1-MAF\right)\times \text{bet}a^{2}$$

Above-mentioned *MAF* means the effect allele frequency and *beta* is the effect estimated of the genetic variant of exposure.

F-statistics representing the strength of correlation between IVs and phenotype was calculated by the following equation:


$$F=\left(R^{2}\times \left(N-2\right)\right)/\left(1-R^{2}\right)$$

Above-mentioned *R^2^* is the proportion of exposure accounted for the genetic variant, and N means the sample size of exposure to assess the strength of the extracted instruments (25).

## 3. Results

### 3.1. Instrument strength

After harmonizing SNPs of each fatty acids traits with GWAS datasets of AD, we, respectively, obtained 47–53 genome-wide SNPs for the fatty acids traits (Supplementary Table S1). The median F statistic, the measurement used to assess instrument strength, was 53.7 (in the range of 23.9–6243.5), indicating that all instruments used in the MR analyses were strong (the recommended F statistic is >10).

### 3.2. Univariable Mendelian randomization

The primary MR analyses indicated that there is a casual link between increased level of n-3 fatty acids and a reduced risk of AD (IVW odds ratio [OR]: 0.92, 95% confidence interval [CI]: 0.87–0.98) (Table 1). Besides, DHA, an essential n-3 fatty acids, could genetically reduce the risk of AD (IVW OR: 0.91, 95% CI: 0.84–0.98) (Supplementary Table S2).

**Table 1:** Univariable causal effects of fatty acids on atopic dermatitis.

Exposure	Outcome	No. SNPs	MR method	*P*	OR	Lower 95% CI limit	Upper 95% CI limit
Omega-6 fatty acids	atopic dermatitis	51	IVW	0.27	0.96	0.90	1.03
			Weighted median	0.82	1.01	0.92	1.11
			Weighted mode	0.66	1.03	0.91	1.16
			MR Egger	0.80	0.98	0.85	1.13
Omega-3 fatty acids		48	IVW	0.01	0.92	0.87	0.98
			Weighted median	<0.001	0.90	0.85	0.95
			Weighted mode	0.003	0.91	0.86	0.97
			MR Egger	0.08	0.92	0.85	1.01
Polyunsaturated fatty acids		53	IVW	0.08	0.94	0.87	1.01
			Weighted median	0.69	0.98	0.89	1.08
			Weighted mode	0.67	1.03	0.91	1.16
			MR Egger	0.89	0.99	0.86	1.14
Saturated fatty acids		47	IVW	0.25	0.95	0.87	1.04
			Weighted median	0.51	1.03	0.94	1.14
			Weighted mode	0.56	1.03	0.93	1.15
			MR Egger	0.45	1.06	0.91	1.23
Monounsaturated fatty acids		56	IVW	0.37	0.96	0.88	1.05
			Weighted median	0.27	0.95	0.87	1.04
			Weighted mode	0.84	0.99	0.90	1.09
			MR Egger	0.87	1.01	0.88	1.17

MR leave-one-out sensitivity analyses demonstrated that the IVW results was significantly influenced by specifical SNP (rs174546) in the analyses of n-3 fatty acids (Supplementary Figure S1). Estimates resulting from Wald ratio method for the single-SNP analyses revealed that rs174546 are likely to play a crucial role in the casual link between n-3 fatty acids levels and AD (rs174546 on n-3 fatty acids OR: 0·90, 95% CI: 0·85–0·96) (Table 2).

**Table 2:** Causal effects of omega-3 fatty acids on AD *via* the *FADS* gene cluster.

Exposure	Outcome	NO. SNPs	MR method	*P*	OR	Lower 95% CI limit	Upper 95% CI limit
Omega-3 fatty acids	Atopic dermatitis	1	Wald ratio	7.64E-04	0.90	0.85	0.96

### 3.3. Pleiotropy and heterogeneity analysis

There was no proof of horizontal pleiotropy found using by MR-egger (Supplementary Table S3), indicating robust relationships between the five fatty acids and AD.

Besides, we also evaluated genetic instrument heterogeneity indicating pleiotropic effects. After removing the pleiotropic instruments detected by MR-PRESSO, the effects of n-3 fatty acids on AD were still robust (Table 3) (OR [95% CI] 0.93[0.88, 0.98] and PIVW = 8.55 × 10^−3^ for n-3 fatty acids).

**Table 3:** Univariable causal effects of omega-3 fatty acids on atopic dermatitis after removing the identified outliers.

Exposure	Outcome	No. SNPs	MR method	*P*	OR	Lower 95% CI limit	Upper 95% CI limit
Omega-3 fatty acids	Atopic dermatitis	47	MR Egger	0.02	0.91	0.84	0.98
			Weighted median	5.90E-04	0.90	0.85	0.96
			IVW	8.55E-03	0.93	0.88	0.98
			Weighted mode	1.64E-03	0.91	0.86	0.96

### 3.4. Multivariable Mendelian randomization

IVW MVMR analyses was used to estimate the independent casual effect of n-3 fatty acids levels on AD conditioned on n-6 fatty acids, MUFAs and SFAs levels. And then, we compared the independent effect of n-3 fatty acids levels on AD resulting from IVW MVMR to the univariable IVW estimates (n-3 fatty acids univariable OR: 0.92, 95% CI: 0.87–0.98; n-3 fatty acids multivariable OR: 0.86, 95% CI: 0.75–0.99). Effect estimates from MVMR analysis of n-3 fatty acids were consistent with the primary analyses (Table 4), which certainly suggesting that n-3 fatty acids have a protection effect on AD.

**Table 4:** Multivariable Mendelian randomization analyses estimating the direct effects of omega-3 fatty acids conditioning on other fatty acids.

Exposure	Outcome	No. SNPs	*P*	OR	Lower 95% CI limit	Upper 95% CI limit
Monounsaturated fatty acids	Atopic dermatitis	92	0.34	0.82	0.54	1.24
Omega-3 fatty acids			0.04	0.85	0.73	0.99
Omega-6 fatty acids			0.26	1.26	0.85	1.86
Saturated fatty acids			0.71	1.14	0.57	2.31

## 4. Discussion

In this study, MR was used to identify the casual link between each kind of fatty acids and AD. We observed that a higher level of the n-3 fatty acids closely associated to a decreased chance of developing AD, suggesting a protective effect of the n-3 fatty acids, including DHA, in AD. MVMR analyses also demonstrated that the independent causal relationship between n-3 fatty acids and AD, indicating that the effects of n-3 fatty acids are probably independent of (and without being mediated or confounded by) n-6 fatty acids, PUFAs or SFAs.

In the MVMR analyses, the *p* value of direct effects of n-3 fatty acids on AD were less significant, most likely because these conditional analyses had less power. Given that the conditioned estimate for n-3 fatty acids was relatively unchanged, it appears that the biosynthetic pathway for n-3 fatty acids may be significant in relation to the risk of AD.

The leave-one-out analyses showed that these effects for n-3 fatty acids were mainly driven by one SNP (rs174546) which is in the *FADS* gene cluster, thus we hypothesize that this relationship may be considerably mediated by desaturation steps occurring in PUFA biosynthesis. Our research offers meaningful information for identifying new biomarkers and comprehending the pathophysiological mechanisms underlying AD.

In real world, it is hard to ensure how fatty acids related to AD. For example, Fujii, M. et al. reported that deficiency of PUFAs is mainly responsible for AD (26). Higher prenatal n-6 fatty acids exposure may raise the odds of AD in a child with maternal inheritance (27). According to some studies, using black currant seed oil, which is with high amounts of EFAs, as a dietary supplement may help to prevent AD (28).

In the absence of trials, it is therefore difficult to determine if changes at any stage along the fatty acids manufacturing pathway would lead to decreased levels of n-3 fatty acids or increasing amounts of particular fatty acids. For instance, dysfunction of essential enzymes that mediate the synthesis of fatty acids will result in the unstable ratio of these fatty acids. Therefore, a chronic pro-inflammatory state could result from relative disordered lipid metabolism (29). N-6 and n-3 fatty acid have a relationship of competitive inhibition, so it is hard to say which has the great influence on AD. Recent studies reported that low-ratio n-6/n-3 fatty acids supplementation significantly decreased the levels of certain inflammation cytokines (30), indicating the importance of alteration in fatty acids synthesis pathway. Meanwhile, our results reveal the importance of rs174583 in fatty acids desaturase 2 (*FADS2*) gene for the regulation of fatty acids in AD. Genetic variation in the FADS locus was strongly associated with biologically important lipids (31). It is known that FADS gene polymorphisms are related with the amounts of fatty acids in human tissues, which may modify how fatty acid-related atopic diseases exist symptoms (32). While the mechanics of actions of n-3 fatty acids on AD are probably complex, identifying independent impacts of the *FADS* gene could illuminate mechanistic pathways in AD etiology.

MR studies are also useful for informing which supplements may have the greatest risk-related effects. As indicated by our study, protective effects of PUFA were demonstrated, with significant indication for the n-3 fatty acids, particularly DHA. Since the exploration for PUFA and AD are continually developing, MR research could provide more insight on which PUFA should be noticed.

The estimated effects of n-3 fatty acids on AD using MR method should be explanatory. Actually, effects discovered using MR mainly resulting from genetic inheritance. Therefore, in traditional traits, the adulthood gained fatty acids supplements over a relatively short period of time are unlikely to have any significant effect. Although our study provides evidence that a higher level of n-3 fatty acids may reduce the chance of developing AD, A placebo-controlled study found no effect of EFAs supplements in AD (33). Kong WS et al. identified the long-chain saturated fatty acids milk diet would develop eczema, accompanied by increased gut type 3 innate lymphoid cells (34). Another cohort study showed that the lower n-3 fatty acid levels in maternal plasma were associated with the prevalence of atopic disorder (35).

Meanwhile, although tissue and blood fatty acids have been used as biomarkers for decades (36), the test methods are developing (37, 38). Further studies regarding the normal range of EFAs are required to assist clinical researchers in the relationship of fatty acids and diseases.

Our studies have several strengths. First, MR analysis is used that could not be influenced by reverse causation and confounding in comparison to ordinary traits, and then we used the recently largest GWAS datasets both chosen from European ancestry, thereby ruling out the confounding variable of population. Meanwhile, using F statistics to identify the genetic instrumental variants are strong enough for MR analysis. Finally, there are different methodological approaches have been applied to prove the estimate effects, including univariable Mendelian randomization, multivariable Mendelian randomization and single-SNP analyses.

However, several limitations need to be listed. Firstly, although the F-statistics of the instruments for our univariable analyses were not weak, weak instrument bias may exist in our MVMR analyses. Second, comprehensive analysis of fatty acids and AD was constrained because we can only obtain now available GWAS data. For example, MVMR analyses can be used to estimate the direct effects of each fatty acids independent of others, but there are no datasets could be able to estimate the effect of PUFA: SFA ratio which may be critical underlying AD pathogenesis. And although the largest GWAS of AD up to now was obtained, the clarified information about the patients was not provided. Therefore, there is no design groups about AD’s stage and severity. Third, The GWAS effect sizes were calculated using fatty acids plasma levels, as a result the fatty acids levels in epidermal layer might not be accurately reflected that might more conspicuously influence chance of developing AD. Finally, based on previous studies, the relationship between diet and dermatologic diseases is not clear (39), further studies are necessary to determine whether PUFAs have effects on AD in the real world.

## 5. Conclusion

In this study, we applied MR to estimate the effect of fatty acids on AD. Our results show a genetically protective role of n-3 fatty acids in the development of AD. These results also imply that patients with AD are more likely to have genetic variation in the *FADS* locus. Thus, it is important for researchers and dermatologists to pay more attention on omega-3 fatty acid.

## Data availability statement

Publicly available datasets were analyzed in this study. This data can be found at: https://gwas.mrcieu.ac.uk/

## Author contributions

J-YL wrote the draft manuscript and prepared the data. L-JM revised the manuscript. J-PY and PY designed the manuscript. B-XB designed and revised the manuscript. All authors have contributed to the article and approved the submitted version.

## Funding

This project was supported by the National Natural Science Foundation of China (NSFC) grant number 81872513.

## Conflict of interest

The authors declare that the study was performed without any commercial or financial relationships that could be construed as a potential conflict of interest.

## Publisher’s note

All claims expressed in this article are solely those of the authors and do not necessarily represent those of their affiliated organizations, or those of the publisher, the editors and the reviewers. Any product that may be evaluated in this article, or claim that may be made by its manufacturer, is not guaranteed or endorsed by the publisher.
