# Supplementary figures and images for: Causal effects of fatty acids on atopic dermatitis: A Mendelian randomization study
Source: Front Nutr. 2023 Feb 23;10:1083455. doi: 10.3389/fnut.2023.1083455 (PMC9996175; doi:10.3389/fnut.2023.1083455)

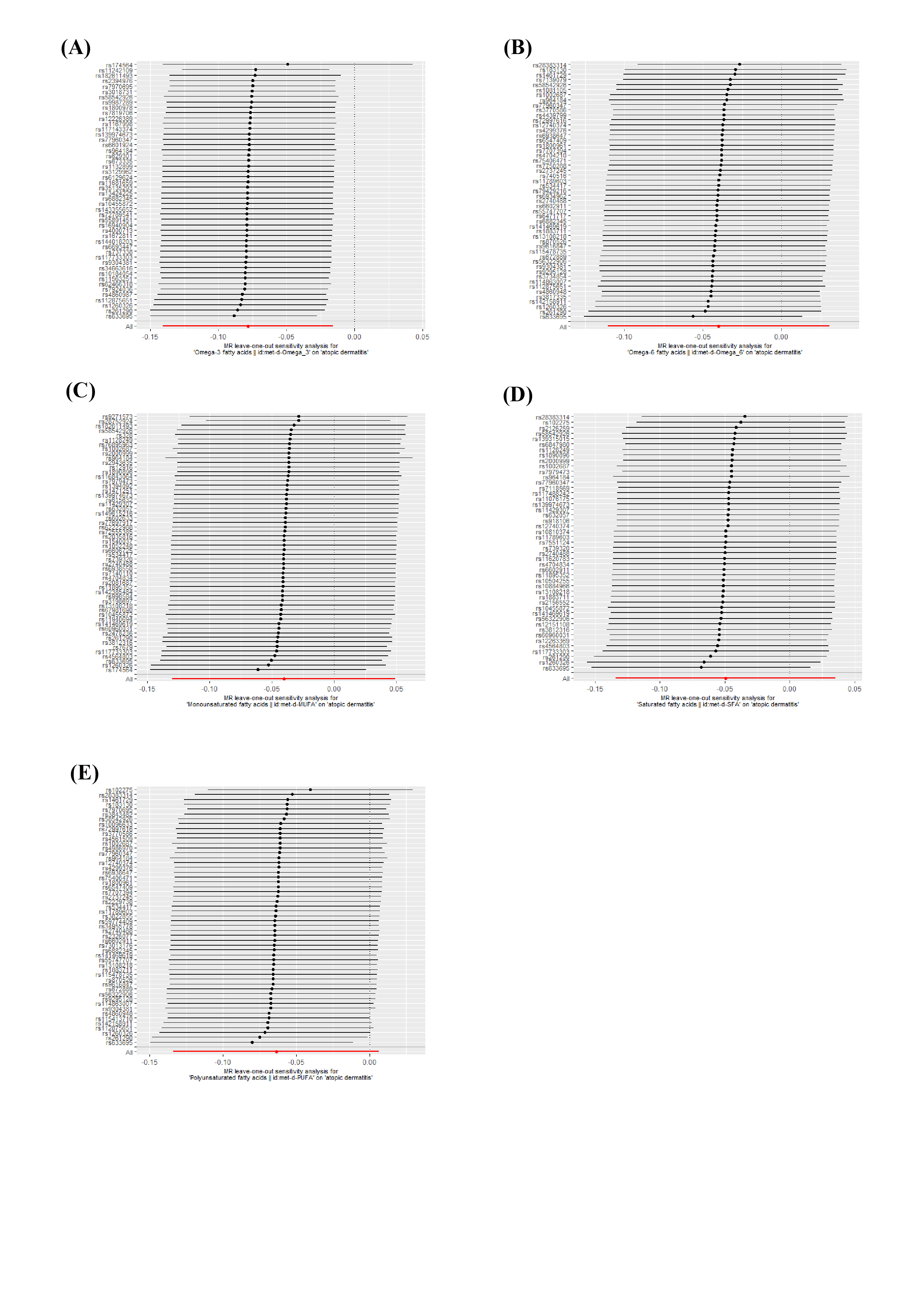

Supplement: Supplementary file 4 [file Image_1.jpeg]
